# Immersive virtual reality on childbirth experience for women: a randomized controlled trial

**DOI:** 10.1186/s12884-022-04598-y

**Published:** 2022-04-23

**Authors:** Elif Gizem Carus, Nazli Albayrak, Halit Mert Bildirici, Selen Gur Ozmen

**Affiliations:** 1 Graduate School of Health Sciences, Neuroscience Master’s Program, Bahcesehir University, Istanbul, Turkey; 2 Department of Obstetrics and Gynecology, School of Medicine, Acibadem Mehmet Ali Aydinlar University, Istanbul, Turkey; 3 American Robert College of Istanbul, Istanbul, Turkey; 4 Department of Physiotherapy and Rehabilitation, Faculty Of Health Sciences, Bahcesehir University, Istanbul, Turkey

**Keywords:** Labor, Immersive Virtual Reality, Pain, Nonpharmacologic Treatment, Anxiety, Depression

## Abstract

**Objective:**

To evaluate the effectiveness of immersive virtual reality (VR) on patient satisfaction as a distractive tool and pain relief among laboring women.

**Methods:**

This was a randomized, controlled clinical trial with 42 laboring women allocated to VR intervention and control groups. Among women in the VR group, patient satisfaction with the use of VR was assessed by a Virtual Reality Satisfaction Survey, measured by a Visual Analog Scale (VAS) score and evaluated by questioning them about whether they would choose VR in future labor. As a primary outcome, patient satisfaction scores regarding the overall childbirth experience were compared between women in the two groups. A secondary outcome was pain assessed by a visual pain rating scale in the early and active phases of labor in women in both groups. Psychometric information was also collected from participants in each group using the Beck Anxiety Inventory and Beck Depression Inventory.

**Results:**

We observed a high level of patient satisfaction with the use of immersive VR during labor. The VAS revealed a mean satisfaction score of 87.7 ± 12.9 out of a maximum of 100. Twenty out of 21 (95%) women in the VR group stated that they would like to use VR again in future labor. VR improved pain scores in early labor and contributed positively to the overall childbirth experience. The mean pain score pre-VR was 2.6 ± 1.2 compared to 2.0 ± 1.3 post-VR (*p* < 0.01). Anxiety and depression scores were similar in participants in the intervention and control groups (*p* = 0.103 and *p* = 0.13, respectively).

**Conclusion:**

Immersive VR application during labor was associated with higher patient satisfaction based on our study findings. VR also improved participants’ pain scores in early labor before epidural administration. Immersive VR may find a place as an adjunct in labor and delivery units to improve lengthy labor experiences for women. Studies with larger groups of participants are needed to confirm these observations.

**Trial Registration:**

ClinicalTrials.gov: NCT05032456

**Supplementary Information:**

The online version contains supplementary material available at 10.1186/s12884-022-04598-y.

## Introduction

Neuraxial blockade, which includes epidural, spinal, and combined spinal-epidural analgesia, is presently the gold standard for pain control in laboring women [1, 2]. However, improving the whole childbirth experience for women is more complex and requires providing individualized care, including alternative treatments.

Opioid and nonopioid pharmacotherapies, patient-controlled analgesia (PCA), and nitrous oxide are all used with variable success for labor pain [3].

Acupuncture, hypnosis, yoga, hydrotherapy, massage, relaxation techniques, and transcutaneous electronic nerve stimulation (TENS) are among the adjuvant treatments provided to women in labor [4–7].

Recent literature indicates the successful use of immersive virtual reality (VR) for a variety of painful medical procedures [8–13]. Via wearing the VR goggles, the user has the illusion of going inside the 3D computer-generated world and visiting novel environments. Immersive VR is hypothesized to reduce pain through distraction, a nonpharmacologic attentional mechanism. With immersive VR, the user's brain is preoccupied with the flood of information presented by the virtual environment, thus reducing the mind’s ability to process pain signals [14].

We hypothesized that laboring women find immersive VR to be a beneficial tool for their overall childbirth experience. We randomly assigned women in labor admitted to our Labor and Delivery floor to a VR group or a control group.

## Materials and methods

### Study design

This was a randomized, controlled, single-center clinical trial in which we enrolled 42 women admitted during labor. We randomized these women to an immersive virtual reality (VR) or control group following their approval and written consent. The study was approved by the Institutional Ethics Committee of Acibadem Mehmet Ali Aydinlar University (IRB protocol no: 2020–18/07) and registered with clinicaltrials.gov (NCT05032456). The goal of this study was to assess whether immersive VR improved patient satisfaction in laboring women. We assessed patient satisfaction among VR users and compared patient satisfaction scores regarding the overall childbirth experience between the two groups as our primary objective. Our second objective was to assess whether VR provided pain relief in the latent or active phase of labor. We also evaluated anxiety and depression in both groups on admission as potential confounders. The study took place at Acibadem Maslak Hospital, a private hospital affiliated with Acibadem University School of Medicine in Istanbul, Turkey. Enrollment completed between November 2020 and June 2021.

### Study subjects

Participants in this study were primigravida or multigravida presenting with labor who were candidates for vaginal birth with no known risk factors. The inclusion criteria were women between 18–42 years of age at 37–41 weeks gestation with a singleton pregnancy, vertex presentation, no history of chronic medical conditions, absence of pregnancy complications, and admission with documented labor by cervical exam and regular uterine contractions. Women with a diagnosis of migraine, headache, dizziness, motion sickness, epilepsy, psychiatric disorders, visual or auditory disabilities, or history of cesarean section were excluded.

Pregnant women between 35 and 37 weeks gestation who were followed by our Obstetric Outpatient Clinic at Acibadem Maslak Hospital were provided information leaflets for our VR study by one of the authors (NA). We have ‘Preparation to birth’ classes for expecting couples one every 6 weeks, and during each of those courses, NA briefly introduced our study, its objectives and handed information leaflets to attendants.

#### VR group

**Fig. 1:**
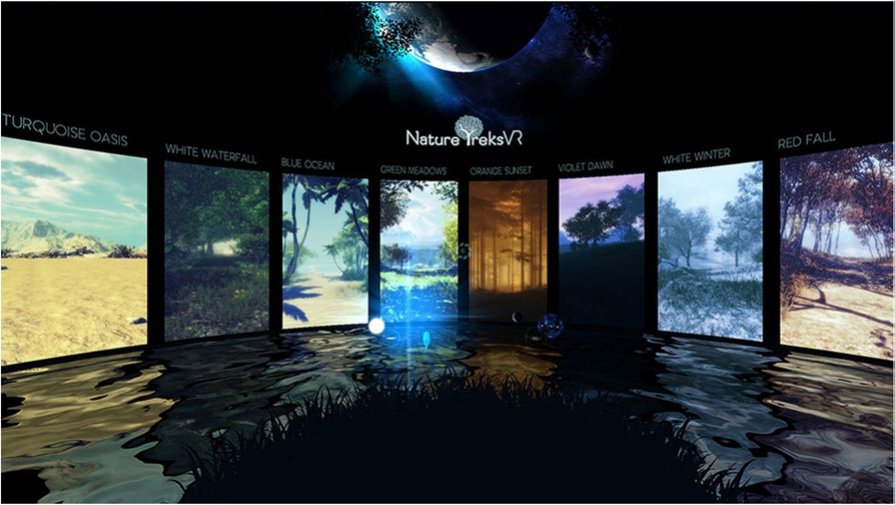
Natural Treks environments

We used an Oculus Quest All-in-one VR Gaming Headset (128 GB) VR system. Before the intervention, the authors introduced the equipment and instructed study participants on how to wear and activate the headsets. Anxiety and depression scales were also applied on admission. The laboring women who enrolled in the VR group first wore the headsets in early labor (cervical dilation 3 cm) for 20 min. The patients were offered to choose among several virtual environments, including orange sunset, green meadows, black beginning, red savannah, blue deep, blue moon, blue ocean, white winter, and red fall (Fig. 1). Cards printed out from the screenshots of the Nature Treks application representing these novel immersion options were provided to the patients to help them pick up their preferred environment in advance. The second implementation of VR headsets was after epidural analgesia was administered in the active phase of labor (cervical dilation 6–7 cm); this implementation was also for 20 min. After the second intervention, the “Virtual Reality Satisfaction Survey” was applied by the authors. Patients were asked to fill out a visual pain rating scale immediately before and after VR use in early and active labor.

#### Control group

For participants randomized to the control group, VR headsets were not used, and our standard of care in laboring women was followed. Anxiety and depression scales were used to evaluate to each subject on admission. Participants in this group completed a visual pain rating scale both in the latent and active phases of labor.

### Clinical measures

To evaluate the effectiveness of immersive VR in laboring women, we evaluated patient satisfaction with the use of VR among the intervention group. Patient satisfaction with the overall childbirth experience and pain scores were compared between the intervention and control groups.

**Fig. 2:**
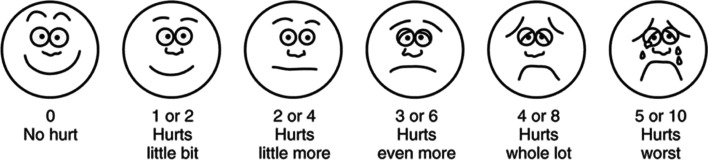
Wong-Baker Faces Pain Scale

Patient satisfaction with the use of VR was assessed by a "Virtual Reality Satisfaction Survey", a 10-question survey prepared by our team and measured with a visual analog scale (VAS) score. 0 being the lowest and 100 being the highest possible VR satisfaction score. We also asked these women whether they would like to use VR in future labor. Patient satisfaction with overall labor and delivery experience was assessed using a numeric rating scale (NRS). All discharged women were called within a week following discharge and asked to rate their overall childbirth experience on a scale from 0 to 10. Zero indicates the most negative experience possible, and 10 indicates the highest satisfaction possible. We classified a score of 8 to 10 as high satisfaction and a score of 4 or less as poor childbirth experience. Pain scores in both early and active labor in each group were assessed using the Wong-Baker Faces Pain Rating Scale [15]. The scale shows a series of 6 faces ranging from a happy face at 0, or "no hurt", to a crying face at 5, which represents "hurts like the worst pain imaginable (Fig. 2).

The anxiety levels of the study participants were assessed with the Beck Anxiety Inventory (BAI) [16]. This inventory consists of 21 items, each scored from 0 to 3. This is a self-report questionnaire measuring somatic and cognitive parts of anxiety. The total score is calculated by finding the sum of 21 items. A score of 0 to 7 indicates minimal anxiety, 8 to 15 indicates mild anxiety, 16 to 25 indicates moderate anxiety, and 30 to 63 is associated with severe anxiety.

For the assessment of depression in each group, the Beck Depression Inventory was used. It consists of 21 items, which is a multiple-choice test and gives a score ranging from 0 to 63. Each answer is scored on a scale value of 0–3. Measures of 0–9 indicate that a person is not depressed, 10–18 indicates mild-moderate depression, 19–29 indicates moderate-severe depression and 30–63 indicates severe depression. This self-rated test estimates the signs of depression, such as pessimism, feelings of failure, self-dissatisfaction, punishment, crying, and insomnia [17, 18].

### Statistical methods

#### Sample size calculation and randomization

A priori power analysis was performed to estimate the sample size with a power (1-β) of 80%, a significance (α) of 0.05 and an allocation ratio of 1. We assumed a neutral satisfaction score of 50 out of 100 (SD = 12.5) for the control group and hypothesized.

25% increase in satisfaction scores with the use of VR. A sample size of 17 subjects per group was computed to observe this difference. For this analysis, G*Power software was used. For potential dropouts, we decided to enroll 21 subjects in each group. We used a random sequence generator (www.random.org), which picked a random sequence of 21 numbers (ranging from 1 to 42) for VR and control columns, respectively. This list was printed and kept in a locked cabinet at the nurses` station of Labor and Delivery floor. Following consent, we asked study participants to pick up a sealed opaque envelope, each containing a number from 1 to 42. Our charge nurse matched this number to the list of numbers for VR or the control group determining their assignment. This method helped to conceal the allocation sequence and provided that an equal number of subjects (*n* = 21) were randomized to the intervention (VR) and control groups. Since the intervention group wore VR headsets and the control group did not, blinding was not possible.

#### Data analysis

Statistical analyses were performed using IBM SPSS Statistics for Windows, Version 25.0. Armonk, NY: IBM Corp. Descriptive statistics are presented as the mean score, standard deviation and absolute frequency. The Shapiro–Wilk test was used to analyze whether the continuous variables followed a normal distribution. To compare the mean overall childbirth satisfaction scores as well as anxiety and depression scores between the two groups, an independent t test or Mann–Whitney U test was used where appropriate. We used a paired samples t test or Wilcoxon t test where appropriate for the comparison of pain scores before and after the VR experience in early and active labor. A p value of less than 0.05 was considered statistically significant.

## Results

**Fig. 3:**
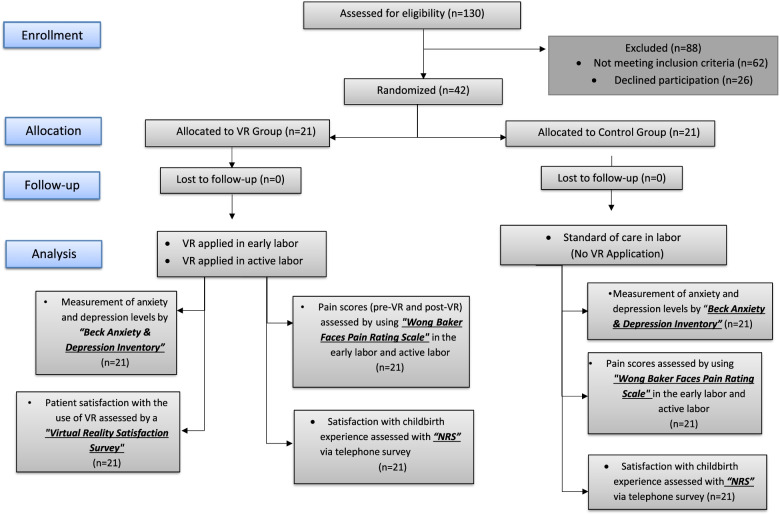
Flowchart of the study

Of the 130 women admitted in labor, 68 were eligible for inclusion in the study, and 42 agreed to participate in this study (Fig. 3). The characteristics of laboring women randomized to either the immersive VR or the control group are shown in Table 1. There was no difference between the two groups in terms of these baseline characteristics.

**Table 1:** Characteristics of the Study Subjects

	VR GROUP (*n* = 21)	CONTROL GROUP (*n* = 21)
Age, Mean ± SD	31.0 ± 2.6	31.8 ± 3.6
Gestational age, Mean ± SD	39.5 ± 0.6	39.0 ± 1.0
Body Mass Index (BMI), Mean ± SD	26.2 ± 2.7	26.7 ± 3.1
Parity		
Primiparous (n) (%)	18(86%)	17(81%)
Multiparous (n) (%)	3(14%)	4(19%)
Delivery Type		
Vaginal Birth (n) (%)	18(86%)	18(86%)
Cesarean Section (n) (%)	3(14%)	3(14%)

All women in the VR group stated they enjoyed the VR experience reflected by the Virtual Reality satisfaction survey. The VAS revealed a mean satisfaction score of 87.7 ± 12.9 out of a maximum of 100. Twenty out of 21 (95%) women in the VR group stated that they would like to use VR again in future labor.

VR intervention improved pain scores in early labor before epidural administration. The mean pain score pre-VR was 2.6 ± 1.2 compared to 2.0 ± 1.3 post-VR (*p* < 0.01). Following epidural analgesia, pain scores were significantly lower, with a mean pain score of 0.8 ± 0.5, which remained unchanged (0.8 ± 0.5) post-VR.

Within one week following discharge, we conducted a telephone survey where we found a significant difference when patient satisfaction with the overall childbirth experience was questioned in the VR and control groups. The NRS score of women in the VR group was 8.8 ± 1.1 compared to 7.9 ± 1.6 in the control group (*p* = 0.04).

The Beck Anxiety Inventory revealed similar anxiety scores in the VR (11.2 ± 7.5) and control groups (7.5 ± 6.7) (*p* = 0.103). The Beck Depression Inventory also revealed similar depression scores in women in the VR (7.6 ± 5.8) and control groups (5.0 ± 5.0) (*p* = 0.13).

Women in the VR group tolerated the VR application very well. No significant adverse events were observed requiring exclusion from the study.

## Discussion

We found that the use of immersive VR in laboring women was associated with a high level of patient satisfaction and likely served as a distractive tool. There are very limited data regarding the use of VR in laboring women. An earlier study evaluated VR use during episiotomy repair and reported less anxiety in the intervention group [15]. Two recent studies reported reduced labor pain and anxiety with the use of VR [19, 20]. Kist et al. showed that VR provided anxiolysis to laboring women undergoing epidural placement [21]. Hajesmeel-Gohari et al. very recently published a scoping review which included a total of nine VR studies on pregnant women and childbirth, vast majority finding VR useful in reducing anxiety and pain [22]. In our study, we also noticed an improvement in pain scores following the use of VR in early labor before epidural administration. Epidural administration afterward obviously provided substantial pain relief, as evidenced by dramatically decreased pain scores in both groups. It is important to clarify that we did not test VR as a substitute for epidural analgesia. Laboring women both in the intervention and control groups received epidural analgesia at their request as part of our standard of care. Our study is the first to assess whether VR might be used as an adjunct to improve the childbirth experience of women. Our awareness regarding the importance of a positive childbirth experience and its impact on women’s physical, psychological and social well-being has gradually increased [23–25]. In addition to ensuring maternal and neonatal health and safety, improving patient satisfaction during childbirth has become an integral part of high-quality care, as also underscored by the WHO [26].

The amount of support from caregivers and a quality caregiver-patient relationship are paramount for patient satisfaction during childbirth [27]. Accordingly, many labor and delivery units provide one-on-one nursing support for laboring women if possible. Most laboring women want to be involved in care decisions and demand that their input be respected. Attendance to antenatal classes and having birth plans are encouraged and increase the odds of a positive childbirth experience [28].

Poor childbirth experience, on the other hand, is a complex issue, which is reported more commonly in primiparous women, in cases of induced labor, operative delivery, emergency cesarean, postpartum hemorrhage, low Apgar score, shoulder dystocia, sphincter injury and maternal infection [29]. The VAS was found to be suitable in the assessment of negative birth experiences [30], and our study supports its potential to evaluate positive childbirth experiences as well.

Since labor is associated with severe pain in most women, pain control is very important to improve patient satisfaction [31, 32]. Currently, epidural analgesia is considered the gold standard in labor analgesia, providing the most effective pain relief during childbirth [33, 34]. On the other hand, many women with healthy pregnancies want to minimize medical interventions and seek nonpharmacological ways to cope with labor pain. In addition, they want to be relaxed yet remain active during labor. Many labor and delivery units are equipped to provide warm showers and birth ball exercises to those in labor. Warm showers in labor were associated with pain reduction and contributed to a positive overall experience [35]. Birth ball exercises also provided significant improvements in childbirth self-efficacy and pain [36]. Some units work with professional therapists and offer massage therapies during labor. Massage therapy during labor was associated with pain relief and decreased anxiety as well as shorter labor duration [37].

Fear of childbirth is common, which leads to increasing rates of cesarean on maternal request [38] and contributes to already high cesarean rates in countries such as Turkey [39]. Considering that cesarean rates have remained high in our centers with 24-h epidural analgesia availability, it is certain that we need to improve the overall childbirth experience beyond pain relief. Our findings of high patient satisfaction with VR use in labor along with routine epidural administration on demand further support this point. We need to present several options to laboring women to choose from, such as warm showers, birth ball exercises, yoga, massage, etc., possibly in an alternating fashion. Our study suggested that immersive VR might also be a valuable tool to distract and potentially entertain women during long hours of labor. VR may especially be instrumental during intermittent fetal monitoring episodes when most of the abovementioned activities are not feasible.

VR users may experience adverse effects, including headache, dizziness, motion sickness, and blurry vision. Immersive VR may also negatively affect static balance [40, 41]. Women in our study did not experience any significant side effects. This might be secondary to our small study size as well as our exclusion of all subjects with a diagnosis of migraine, headache, dizziness, motion sickness, and epilepsy. It is also worth mentioning that prior to this study, we conducted a survey among several volunteer pregnant women, and the vast majority indicated that they preferred immersion in nature (Nature Treks environments) compared to other options, including playing games.

Although randomization and strict adherence to the standard of care in both groups of laboring women are strengths of our study, it has several limitations as well. The numbers are relatively small, and obviously larger studies are needed to support our findings. It is also worth mentioning that this study was performed at a single upscale health center serving an affluent cohort potentially biased toward newer technology such as VR.

## Conclusion

Based on the results of this preliminary study, immersive VR may improve pain scores likely through distraction in the latent phase of labor prior to epidural administration. VR might also improve patient satisfaction, an important indicator for the quality of care in childbirth.

## Supplementary Information

**Additional file 1. **

## Data Availability

All data generated/analysed during the current study are provided as a supplementary information file.

## Abbreviations

VR: Virtual Reality:
PCA: Patient-Controlled Analgesia
NRS: Numerical Rating Scale:
BAI: Beck Anxiety Inventory
BDI: Beck Depression Inventory
WHO: World Health Organization
